# Sensitivity, specificity, and cutoff points of the Cough Severity Index (CSI-Br) and the Newcastle Laryngeal Hypersensitivity Questionnaire (LHQ-Br) in individuals with chronic refractory cough

**DOI:** 10.1590/2317-1782/e20250213en

**Published:** 2026-07-10

**Authors:** Camila Moura Menezes, Rodrigo Dornelas, Mara Behlau, Vanessa Veis Ribeiro

**Affiliations:** 1 Programa de Pós-graduação em Ciências Médicas – PPGCM, Universidade de Brasília – UnB - Brasília (DF), Brasil.; 2 Universidade Federal do Rio de Janeiro – UFRJ - Rio de Janeiro (RJ), Brasil.; 3 Centro de Estudos da Voz – CEV - São Paulo (SP), Brasil.

**Keywords:** Self-assessment, Speech-Language, Signs and Symptoms, Chronic Cough

## Abstract

**Purpose:**

To establish cutoff points and evaluate the discriminative accuracy of the self-perception instruments Cough Severity Index (CSI-Br) and Newcastle Laryngeal Hypersensitivity Questionnaire (LHQ-Br) in identifying symptoms related to chronic refractory cough (CRC) in adults.

**Methods:**

This is a cross-sectional, observational, quantitative study with a sample of 157 participants (111 females and 46 males), with a mean age of 44.92 years. They were divided into two groups based on the presence or absence of CRC: Group with CRC (CRC-G) with 110 participants (31 males and 79 females), with a mean age of 48.67 (SD: 16.04) years; The study included a healthy control group (CG) with 47 participants (15 males and 32 females), with a mean age of 36.34 (SD: 14.31) years. Data were collected in person at the outpatient clinics of the participating hospitals and online via Google Forms. Participants answered the CSI-Br and LHQ-Br. Analysis was performed using SPSS 29.0 software, employing ROC curves to determine cutoffs and performance metrics.

**Results:**

For the LHQ-Br, the area under the curve (AUC) was 0.753, with a cutoff of 5.8, sensitivity of 70%, and specificity of 73.4%. The CSI-Br had an AUC of 0.894 in the total score, with a cutoff of 4.5, sensitivity of 84.9%, and specificity of 83%.

**Conclusion:**

Based on the established cutoffs, both questionnaires proved to be sensitive and specific, with greater robustness for CSI-Br.

## INTRODUCTION

Coughing is a complex reflex that can become chronic under certain clinical conditions^(1-3)^. It manifests differently depending on its duration, being called a chronic cough (CC) when it persists for more than 8 weeks. In these cases, the cough ceases to be beneficial to the body and can lead to health problems^(1,3)^. CC, in the absence of other underlying clinical factors, is mainly characterized by a worsened cough reflex. It is not fully understood, but it may be correlated with peripheral and central mechanisms^(3-5)^.

When CC is refractory to medical treatment for traditional causes, it is called chronic refractory cough (CRC)^(6,7)^. Cases of CRC may have a primary focus in the larynx, presenting clinical characteristics such as abnormal sensation in the throat (laryngeal paresthesia), increased sensitivity to cough in response to known tussigens (hypertussia), and cough triggered in response to non-tussigenic stimuli (allotussia)^(3)^. In addition, these patients may commonly present dysphonia as a co-causal factor or associated with CRC^(3,6-8)^.

CRC is associated with central and peripheral mechanisms, involving hypersensitivity of laryngeal receptors and alterations in the sensory and behavioral processing of cough^(3)^. From this perspective, CRC may present a learned and maintained response, characterized as a maladaptive behavioral response. Thus, speech-language-hearing management aimed at modulating the conscious response to tussigens that trigger CRC is a possible treatment^(7,9)^.

This requires an adequate speech-language-hearing assessment, using objective and subjective procedures^(10)^. Although international literature reports objective monitoring devices for CRC frequency and intensity^(11)^, they are not commercially available in Brazil. This reinforces the importance of subjective self-assessment instruments^(12-15)^.

Self-assessment measures the patient's perception of the cough, the symptoms, and the discomfort caused in the patient's daily life, which cannot be measured by other types of assessment^(10,12-17)^. Specifically for cases of CRC, it is even more relevant in the Brazilian context to have accessible and nationally validated self-assessment instruments for speech-language-hearing clinical practice.

Currently, there are only two validated cough self-assessment instruments in Brazilian Portuguese (BP), related to different constructs^(12-15)^. The Cough Severity Index (CSI-Br)^(12,13,16)^ can be used to measure self-perception of the severity of cough symptoms, and the Newcastle Laryngeal Hypersensitivity Questionnaire (LHQ-Br)^(14,15,17)^ can be used to estimate self-perception of sensations and symptoms related to laryngeal hypersensitivity. Although the CSI-Br and LHQ-Br have already been validated for BP^(12-15)^, their cutoff points and discriminatory power in individuals with CRC are not yet known, within the constructs that each instrument proposes to evaluate.

The cutoff allows for the classification and clinical interpretation of the results. Thus, defining their cutoffs will allow clinicians to transform the scores into useful information, facilitating the interpretation of each patient's results and guiding more precise therapeutic decisions^(18,19)^. They can also be used to monitor the evolution of interventions.

This study aimed to establish cutoff points and evaluate the discriminative accuracy of the CSI-Br and LHQ-Br self-perception instruments in identifying symptoms related to CRC in adults.

## METHODS

This observational, cross-sectional, quantitative, interinstitutional research was approved by the Research Ethics Committee (CEP) of the institution of origin under approval number 4,638,335. The study was conducted in person at the outpatient clinic of the Clementino Fraga Filho University Hospital of the Federal University of Rio de Janeiro, at the Lauro Wanderley University Hospital of the Federal University of Paraíba, and online via Google Forms. Participants were recruited between 2020 and 2024 through the distribution of an invitation folder with eligibility criteria and researcher contacts, in person at the collection hospitals, and through digital media.

Individuals who agreed to participate signed an informed consent form and answered a screening questionnaire with objective individual questions about the research eligibility criteria. The research complied with Resolution No. 466/2012 of the Brazilian National Health Council. The study selected Brazilians, speakers of BP, aged 18 to 65 years, of both sexes, with and without complaints of cough and a medical diagnosis of CRC. They were divided into two groups: the CRC Group (CRC-G) and the healthy control group (CG). The inclusion criteria for the CRC-G were age over 18 years, self-reported persistent cough for more than 8 weeks, refractory to medical treatment, and a medical diagnosis of CRC. For the CG, the criteria were age over 18 years, self-reported good general health status, without complaints or self-reported history of persistent cough, dysphonia, or gastric disease. The exclusion criteria for both groups were individuals with a self-reported medical history of neurological, cognitive, or psychiatric disorders that would limit their understanding of the instructions.

The sample size was estimated based on the population size. The parameters used were population size (N) of 7,939,386 (8% of 99,242,314), with a 10% margin of error (e), a 95% confidence level (z = 1.96), and a population proportion of individuals in the studied category (p) of 0.5. A sample size (n) of 97 participants was calculated. The sample for this study consisted of 157 participants aged 18 to 65 years, with a mean age of 44.92 years, of whom 111 were female, and 46 were male. The CRC-G had 110 participants, of which 31 were male, and 79 were female, with a mean age of 48.67 (SD: 16.04) years; the CG had 47 participants, of which 15 were male, and 32 were female, with a mean age of 36.34 (SD: 14.31) years.

Data collection included a self-report item on the presence of CRC, and the application of the CSI-Br and LHQ-Br self-assessment questionnaires.

CC self-perception was used as a reference for establishing the cutoffs. It consisted of a self-reported cough lasting more than 8 weeks, persistent despite medical treatment and unrelated to other health conditions (clinical picture of CRC)^(3)^.

The CSI-Br is validated in BP^(12,13)^, composed of 10 items, which measure the severity of CC symptoms and their influence on different aspects of the patient's life^(16)^. The instrument has a response key with a 5-level Likert-type scale, ranging from 0 (never) to 4 (always). The instrument has two factors: physical and social activities, composed of items 2, 3, 4, 5, 8, and 10; and functional and psychological, composed of items 1, 6, 7, and 9. The higher the CSI-Br score, the greater the patient's perception of the severity of the cough and the impact of the symptoms on quality of life, with a worse self-perceived health condition^(16)^.

The LHQ-Br is a self-perception instrument of laryngeal sensations associated with laryngeal hypersensitivity^(17)^, validated in BP^(14,15)^. It has 12 items and a Likert-type scale response key ranging from 1 (always) to 7 (never). The LHQ-Br has only one factor, calculated by averaging the 12 items. The higher the LHQ-Br score, the lower the perception of laryngeal sensations. Thus, the lower the score, the worse the patient's cough condition^(17)^.

The statistical software used was IBM SPSS 29.0. The cutoff was determined by analyzing the area under the curve (AUC) using the receiver operating characteristic (ROC) curve, based on the relationship between the highest sensitivity and specificity values^(13,14)^. The ROC curve measures the discriminatory capacity of a test – i.e., its ability to distinguish between individuals with and without a specific condition. The cutoff was evaluated as a classifier using the maximum Kolmogorov-Smirnov (KS) test and the Gini index. The AUC ranges from 0 to 1; values ​​above 0.7 are considered good, between 0.71 and 0.8 are excellent, and below 0.69 indicate zero discrimination^(13,14)^.

## RESULTS

**Figure 1:**
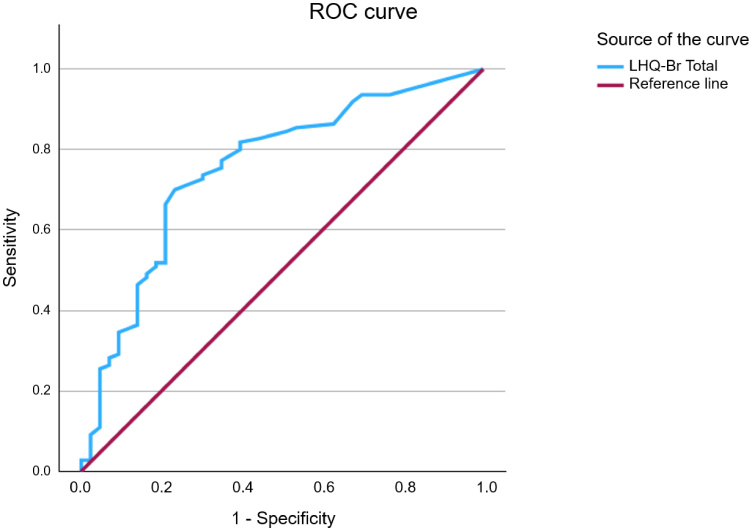
ROC curve of the Newcastle Laryngeal Hypersensitivity Questionnaire (LHQ-Br)

The LHQ-Br in the total score presented an AUC of 0.753 (95% CI: 0.666 to 0.841; p < 0.001), as shown in Figure 1 and Table 1.

**Table 1:** ROC curve of the Newcastle Laryngeal Hypersensitivity Questionnaire (LHQ-Br)

Variable	Area	Test statistics	p-value	95% CI	
				Lower limit	Upper limit
LHQ-Br	0.753	0.045	0.000	0.666	0.841

Caption: CI = confidence interval; LHQ-Br = Newcastle Laryngeal Hypersensitivity Questionnaire

The LHQ-Br also obtained a Gini index of 0.507 and a KS metric of 0.467, with the suggested cutoff of 5.8 and a model quality of 0.67. This cutoff presents a sensitivity of 70.0% and a specificity of 73.4% (1 – specificity = 0.266), resulting in a Youden index of 0.467 (Table 2).

**Table 2:** ROC curve coordinates of the Newcastle Laryngeal Hypersensitivity Questionnaire (LHQ-Br)

Variable	Positive if greater than or equal to	Sensitivity	1 – Specificity	Youden Index
LHQ-Br	5.8	0.700	0.266	0.467

Caption: LHQ = Newcastle Laryngeal Hypersensitivity Questionnaire

**Figure 2:**
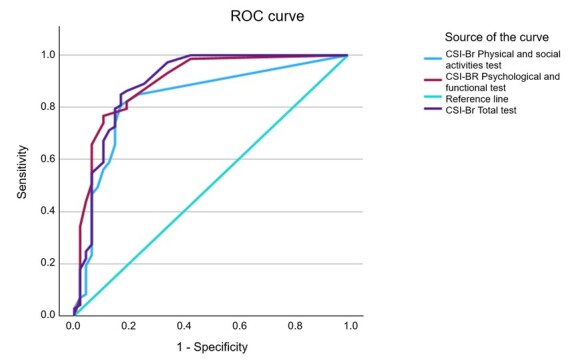
ROC curve of the Cough Severity Index (CSI-Br)

The CSI-Br in the total score presented an AUC of 0.894 (95% CI: 0.827 to 0.960; p < 0.001). The physical and social activities factor had an AUC of 0.832 (95% CI: 0.752 to 0.912; p < 0.001), and the psychological and functional factor had the highest AUC among the scores analyzed, with a value of 0.898 (95% CI: 0.837 to 0.959; p < 0.001). These results can be seen in Figure 2 and Table 3.

**Table 3:** Area under the ROC curve of the Cough Severity Index (CSI-Br)

Variable	Area	Test statistics	p-value	95% CI	
				Lower limit	Upper limit
CSI-Br Total	0.894	0.034	0.000	0.827	0.960
CSI-Br Physical and social activity	0.832	0.041	0.000	0.752	0.912
CSI-Br Psychological and emotional	0.898	0.031	0.000	0.837	0.959

Caption: CI = confidence interval; CSI-Br = Cough Severity Index

The CSI-Br Total obtained a Gini index of 0.787 and a KS metric of 0.679, with the suggested cutoff of 4.50 and a model quality of 0.83. This cutoff has a sensitivity of 84.9% and a specificity of 83.0%, with a Youden index of 0.679. The CSI-Br Physical and Social Activities factor showed a Gini index of 0.663 and a KS metric of 0.638, with a cutoff of 1.50 and a model quality of 0.84. This cutoff point presents a sensitivity of 80.8% and a specificity of 83.0%, resulting in a Youden index of 0.638. The CSI-Br Psychological and Functional factor had a Gini index of 0.796 and a KS metric of 0.661, with a cutoff of 4.50 and a model quality of 0.75. This cutoff obtains a sensitivity of 76.7% and a specificity of 89.4%, with a Youden index of 0.661 (Table 4).

**Table 4:** ROC curve coordinates of the Cough Severity Index (CSI-Br)

Variable	Positive if greater than or equal to	Sensitivity	1 – Specificity	Youden Index
CSI-Br Total	4.50	0.849	0.170	0.679
CSI-Br Physical and social activity	1.50	0.808	0.170	0.638
CSI-Br Psychological and emotional	4.50	0.767	0.106	0.661

Caption: CSI-Br = Cough Severity Index

## DISCUSSION

Speech-language-hearing clinical assessment of CRC uses clinical tests, self-assessment procedures, and complementary vocal assessments^(10)^. Self-assessment is important among these procedures, since it measures data that cannot be obtained by any other form of clinical assessment, in addition to showing the influence of the clinical picture on the patient's daily life^(12,14)^. Therefore, it has gained more space in speech-language-hearing assessment, being clinically considered an important assessment to plan management and analyze the possibility of speech-language-hearing discharge for CRC patients.

The validation of the CSI-Br^(13)^ and LHQ-Br^(15)^, combined with the determination of their respective cutoffs, reveals these tools’ ability to discriminate between individuals with and without CRC. This characteristic is especially relevant in the context of evidence-based practice, as it allows health professionals to use valid and specific instruments to assess the severity of symptoms and the functional impact of CRC on patients' lives^(18,19)^.

It is important to revisit the constructs of the instruments to understand their cutoffs: the CSI-Br^(12,13)^ assesses the perception of the impact of CC on the person's life^(16)^, and the LHQ-Br^(14,15)^ assesses the perception of symptoms related to laryngeal hypersensitivity^(17)^.

The AUC ranges from 0 to 1 – values ​​between 1.0 and 0.9 are considered excellent^(20)^, between 0.9 and 0.8, good^(20)^, between 0.8 and 0.7, fair^(20)^, between 0.7 and 0.6, weak^(20)^, and below 0.5, a failure (i.e., it does not discriminate better than chance)^(20)^. The LHQ-Br total score presented an AUC with fair values^(20)^, while the CSI-Br was considered good in all factors and in the total score; the physical and functional factor had the highest AUC among the measures.

According to these indices, both instruments had acceptable values, but the CSI-Br showed more robust performance in all metrics analyzed, demonstrating better discriminative capacity. The instrument's specific factors, "physical and social activities" and "psychological and functional," provide a multidimensional view of the impact of CRC, which can assist in planning personalized and individualized interventions. This result corroborates the literature that highlights the importance of self-assessment instruments with specific approaches for different aspects of the lives of patients with CRC^(16,17)^.

The LHQ-Br^(15)^ showed moderate and balanced sensitivity and specificity. Moreover, the Youden, Gini, and KS indices confirmed a model with moderate discrimination. This indicates that its cutoff can be useful for screening, allowing the identification of patients with possible symptoms of laryngeal hypersensitivity, and for diagnostic support, helping to reduce false positives^(21)^. However, its performance should be interpreted as acceptable and complementary, not being used alone to confirm symptoms of laryngeal hypersensitivity in patients.

In turn, the CSI-Br^(13)^ proved to be sensitive and specific. The Youden, Gini, and KS indices confirmed good to excellent discriminative capacity in the total score and subscales, characterizing a consistent performance, classifying the model as good to excellent in all metrics evaluated. It offers an effective cutoff for identifying patients with symptoms compatible with CRC and minimizing false positives. This makes it a useful tool for initial screening of patients with suspected CRC and for supporting the diagnostic process^(21)^. This characteristic is particularly relevant in the clinical context, since the early identification of patients with symptoms compatible with CRC can favor a more assertive therapeutic management.

The sensitivity and specificity values ​​obtained were relatively high and close to each other, being at a moderate level for the LHQ-Br and at a high level for the CSI-Br. This finding is relevant because both perform adequately within the dimensions they propose to evaluate. However, the proximity of the values ​​should not be interpreted as equivalence between the instruments, because they analyze different constructs: the LHQ-Br marks self-perceived laryngeal sensations associated with laryngeal hypersensitivity^(12,13,16)^, and the CSI-Br measures the severity of CC symptoms and their impact on different aspects of the patient's life^(14,15,17)^.

The LHQ-Br, although presenting satisfactory values, had a slightly lower sensitivity and specificity than its original English-validated version^(16)^. This difference can be attributed to the cultural specificities of BP and the differences in establishing the cutoff. The CSI-Br^(13)^ maintained good accuracy in relation to the original English-validated protocol^(16)^, which also reported high AUC values, generally above 0.85, and high sensitivity and specificity indices.

The analysis of combined data, without direct comparison between the instruments, indicated a possible limitation of the LHQ-Br^(15)^ in relation to the CSI-Br^(13)^, namely, its more extensive response key, with a 7-point Likert-type scale. This characteristic differentiates it from most self-assessment instruments used in clinical contexts, which generally adopt more concise scales. This may impact the patients’ understanding and response consistency.

It is also important to consider that this study had a sample composed exclusively of patients with CRC, while the scope of application of the tool for laryngeal hypersensitivity is more comprehensive. This difference may influence the generalization of the findings and highlights the need for future investigations with more heterogeneous populations within the spectrum of laryngeal hypersensitivity.

The LHQ-Br cutoff is 17.1 in the original language^(17)^ and 5.8 in the present study. These divergences may be attributed to the different structuring of the instruments. In the BP validation process^(15)^, two items were eliminated in the LHQ-Br, and the structure was unifactorial. The score, in turn, was calculated by averaging the items^(15)^. The international instrument has 14 items distributed in three factors (laryngeal sensitivity, sensation of discomfort, sensation of pressure)^(17)^. Its score per factor is an average, and the total is the sum of the average of the three factors^(17)^. However, considering that the BP version^(15)^ is unifactorial, dividing the international cutoff of 17.1 by three gives 5.7, a value very similar to the Brazilian cutoff of 5.1.

The CSI-Br has a cutoff point of 3 in the original language^(16)^ and 4.5 in BP. The Brazilian version^(13)^ has 10 items, divided into two factors (physical and social activities, psychological and functional), which reflects a concern with the specific dimensions of quality of life, adapted to the cultural perception of symptoms; in turn, the international version has 10 items treated as a single total score^(16)^. Furthermore, the international cutoff was established based on the average difference between individuals with and without CRC, adding to this value the score of two standard deviations from the mean^(16)^. It is believed that the difference in the procedures for establishing the cutoff justifies the divergence for the cutoff in BP.

This study highlights the relevance of self-assessment in clinical practice, especially in conditions that have significant subjective components, such as CRC. Patients' ability to report their perceptions and experiences regarding symptoms helps to define the diagnosis and allows for a more patient-centered approach. Thus, the definition of cutoffs is an important advance in transforming subjective data into clinically interpretable information, contributing to improved clinical decision-making. Thus, the cutoff points of speech-language-hearing self-assessment instruments also contribute to scientific progress within studies related to CRC.

Although this study has provided robust evidence on the applicability and clinical usefulness of the CSI-Br and LHQ-Br, it is important to consider the specific conditions of the Brazilian context, with limited access to objective diagnostic technologies for CRC. This reality makes the instruments studied even more relevant, but it also poses a limitation, since the reference used was the patient's self-perception and not an objective assessment of the CRC diagnosis. Another study limitation is the uneven distribution between groups, which may reduce the accuracy of the specificity estimate. This limitation was mitigated by presenting 95% confidence intervals, which convey the true uncertainty of the estimates and enable proper interpretation of the findings.

## CONCLUSION

The identified cutoff points were 5.8 for the LHQ-Br and 4.5 for the CSI-Br, with specific values ​​of 1.5 for the physical and social domain, and 4.5 for the psychological and functional domain. The results indicate that the LHQ-Br has moderate applicability for initial screening and diagnostic support of patients with laryngeal sensations associated with laryngeal hypersensitivity. The CSI-Br demonstrated more robust performance, being useful both for initial screening and as a support in the diagnostic process, by measuring the severity of CRC symptoms and their influence on different aspects of the patient's life. These findings reinforce the applicability of both instruments, which contribute differently according to their constructs. Nevertheless, both are relevant for the early recognition and appropriate management of patients with CRC.
