# Supplementary material for: Sensitivity, specificity, and cutoff points of the Cough Severity Index (CSI-Br) and the Newcastle Laryngeal Hypersensitivity Questionnaire (LHQ-Br) in individuals with chronic refractory cough
Source: Codas. 2026 Jul 10;38(4):e20250213. doi: 10.1590/2317-1782/e20250213en (PMC13359253; doi:10.1590/2317-1782/e20250213en)
Supplement: Translation

# Translation: Sensibilidade, especificidade e pontos de corte do Índice de Severidade da Tosse (CSI-Br) e do Questionário Newcastle de Hipersensibilidade Laríngea (LHQ-Br) em indivíduos com tosse crônica refratária

**DOI:** 10.1590/2317-1782/e20250213pt

**Authors:** Camila Moura Menezes, Rodrigo Dornelas, Mara Behlau, Vanessa Veis Ribeiro

**Affiliations:** 1 Programa de Pós-graduação em Ciências Médicas – PPGCM, Universidade de Brasília – UnB - Brasília (DF), Brasil.; 2 Universidade Federal do Rio de Janeiro – UFRJ - Rio de Janeiro (RJ), Brasil.; 3 Centro de Estudos da Voz – CEV - São Paulo (SP), Brasil.

**Keywords:** Autoavaliação, Fonoaudiologia, Sinais e Sintomas, Tosse Crônica

## Abstract

**Objetivo:**

Estabelecer pontos de corte e avaliar a acurácia discriminativa dos instrumentos de autopercepção Índice de Severidade da Tosse (CSI-Br) e Questionário Newcastle de Hipersensibilidade Laríngea (LHQ-Br) na identificação de sintomas relacionados à tosse crônica refratária (TCR) em indivíduos adultos.

**Método:**

Trata-se de um estudo transversal, observacional e quantitativo. A amostra foi composta por 157 participantes, com idade média de 44,92 anos, sendo 111 do sexo feminino e 46 do sexo masculino. Os participantes foram distribuídos em dois grupos a partir da presença ou não de TCR: Grupo com TCR (GT) com 110 participantes, sendo 31 do sexo masculino e 79 do sexo feminino, idade média de 48,67 (DP: 16,04) anos; e, Grupo Controle Saudável (GC) com 47 participantes, sendo 15 do sexo masculino e 32 do feminino, idade média de 36,34 (DP: 14,31) anos. A coleta de dados foi presencial nos ambulatórios dos hospitais participantes e on-line via Google Forms. Os participantes responderam o CSI-Br e o LHQ-Br. A análise foi realizada no software SPSS 29.0, utilizando curvas ROC para determinar pontos de corte e métricas de desempenho.

**Resultados:**

Para o LHQ-Br, a área sob a curva (AUC) foi de 0,753, com ponto de corte de 5,8, sensibilidade de 70% e especificidade de 73,4%. O CSI-Br apresentou AUC de 0,894 no escore total, com ponto de corte de 4,5, sensibilidade de 84,9% e especificidade de 83%.

**Conclusão:**

Conclui-se que a partir dos pontos de corte estabelecidos, ambos se mostraram sensíveis e específicos, com maior robustez para o CSI-Br.

## INTRODUÇÃO

A tosse é um reflexo complexo que pode tornar-se crônico em determinadas condições clínicas^(1-3)^. Ela manifesta-se de forma distinta conforme sua duração, sendo denominada como Tosse Crônica (TC) quando persistente por um período de duração maior que oito semanas. Nesses casos a tosse passa a não ser benéfica para o organismo, podendo acarretar prejuízos à saúde^(1,3)^. A TC, na ausência de outros fatores clínicos subjacentes, é caracterizada principalmente pelo reflexo de tosse exacerbado, e não é totalmente compreendida, podendo haver uma correlação com mecanismos periféricos e centrais^(3-5)^.

Quando a TC é refratária ao tratamento médico para as causas tradicionais, ela passa a ser denominada de Tosse Crônica Refratária (TCR)^(6,7)^. Os casos de TCR podem ter foco primário na laringe, apresentando características clínicas como sensação anormal na garganta (parestesia laríngea), aumento da sensibilidade à tosse em resposta a tussígenos conhecidos (hipertussia) e tosse desencadeada em resposta a estímulos não tussígenos (alotussia)^(3)^. Além disso, esses pacientes podem comumente apresentar disfonia como fator co-causal ou associado à TCR^(3,6-8)^.

A TCR é associada a mecanismos centrais e periféricos, envolvendo a hipersensibilidade dos receptores laríngeos e alterações no processamento sensorial e comportamental da tosse^(3)^. Nessa perspectiva, a TCR pode apresentar uma resposta aprendida e mantida, caracterizando-se como uma resposta comportamental mal adaptativa. Desta forma, o manejo fonoaudiológico voltado para a modulação da resposta consciente ao gatilho que desencadeia a TCR passa a ser uma possibilidade de tratamento^(7,9)^.

Para isso, é imprescindível uma avaliação fonoaudiológica adequada, utilizando procedimentos objetivos e subjetivos^(10)^. Embora dispositivos de monitoramento objetivo de frequência e intensidade do TCR sejam relatados na literatura internacional^(11)^, no Brasil eles não são comercializáveis. Isso reforça a importância da avaliação subjetiva, por meio de instrumento de autoavaliação^(12-15)^.

A autoavaliação permite mensurar a percepção do paciente sobre a tosse, os sintomas e o incômodo causado no dia a dia do paciente, o que não pode ser mensurado por outros tipos de avaliação^(10,12-17)^. Especificamente para os casos de TCR, torna-se ainda mais relevante no contexto brasileiro ter instrumentos de autoavaliação acessíveis e validados nacionalmente para a prática clínica fonoaudiológica.

Atualmente existem apenas dois instrumentos validados em PB para autoavaliação da tosse, relacionados a diferentes construtos^(12-15)^. Para mensurar a autopercepção da severidade dos sintomas de tosse pode-se utilizar o Índice de Severidade da Tosse (CSI-Br)^(12,13,16)^ e para estimar a autopercepção de sensações e sintomas relacionados à hipersensibilidade laríngea, pode-se utilizar o Questionário Newcastle de Hipersensibilidade Laríngea (LHQ-Br)^(14,15,17)^. Embora o CSI-Br e o LHQ-Br já estejam validados para o PB^(12-15)^, ainda não se conhecem seus pontos de corte nem seu poder discriminatório em indivíduos com TCR, dentro dos construtos que cada instrumento se propõe a avaliar.

O ponto de corte permite classificar e interpretar clinicamente os resultados. Assim, a definição de seus pontos de corte permitirá que o clínico transforme os escores em informações úteis, facilitando a interpretação dos resultados de cada paciente e orientando decisões terapêuticas mais precisas^(18,19)^. Além disso, podem ser utilizados para acompanhar a evolução das intervenções.

O objetivo do presente estudo foi de estabelecer pontos de corte e avaliar a acurácia discriminativa dos instrumentos de autopercepção CSI-Br e LHQ-Br na identificação de sintomas relacionados à TCR em indivíduos adultos.

## MÉTODO

Esta pesquisa tem delineamento observacional e transversal, quantitativo, de caráter interinstitucional. Ela foi aprovada pelo Comitê de Ética em Pesquisa (CEP) da instituição de origem sob parecer n° 4.638.335. O estudo foi desenvolvido de forma presencial no ambulatório do Hospital Universitário Clementino Fraga Filho da Universidade Federal do Rio de Janeiro, no Hospital Universitário Lauro Wanderley da Universidade Federal da Paraíba e online na plataforma *google forms*. Os participantes foram recrutados entre 2020 e 2024, por meio de divulgação de folder de convite com os critérios de elegibilidade e contatos dos pesquisadores, presencialmente nos hospitais de coleta e em mídias digitais.

Os indivíduos que concordaram em participar assinaram o Termo de Consentimento Livre e Esclarecido (TCLE) e responderam um questionário de triagem com perguntas individuais objetivas sobre os critérios de elegibilidade da pesquisa. A pesquisa respeitou a Resolução nº 466/2012-CNS.

Foram selecionados para o presente estudo indivíduos brasileiros, falantes do PB, com idades entre 18 e 65 anos, de ambos os sexos, com e sem presença de queixa de tosse e diagnóstico médico de TCR. Esses indivíduos foram distribuídos em dois grupos: Grupo com TCR (GT) e Grupo Controle Saudável (GC). Os critérios de inclusão para o GT foram: idade superior a 18 anos, autorrelato da presença de tosse persistente a mais de oito semanas, refratária ao tratamento médico e diagnóstico médico de TCR. Já para o GC foram: idade superior a 18 anos, autorrelato de bom estado geral de saúde, sem queixas ou história autorreferida de tosse persistente, disfonia ou doença gástrica. Os critérios de exclusão aplicados a ambos os grupos foram: indivíduos com histórico médico autorrelatado de distúrbios neurológicos, cognitivos ou psiquiátricos que tornassem a compreensão das instruções limitada.

Para estimar o tamanho da amostra foi realizado um cálculo amostral pelo tamanho da população. Os parâmetros utilizados foram: tamanho da população (N) de 7.939.386 (8% de 99.242.314), com margem de erro (e) de 10%, nível de confiança de 95% (z=1,96) e proporção populacional de indivíduos da categoria estudada (p) de 0,5. Calculou-se um tamanho amostral (n) de 97 participantes. A amostra do presente estudo foi composta por 157 participantes com idades entre 18 e 65 anos, média de 44,92 anos, sendo 111 do sexo feminino e 46 do sexo masculino. O GT com 110 participantes, sendo 31 do sexo masculino e 79 do sexo feminino, idade média de 48,67 (DP: 16,04) anos, e GC com 47 participantes, sendo 15 do sexo masculino e 32 do feminino, idade média de 36,34 (DP: 14,31) anos.

A coleta de dados incluiu um item de autorrelato de presença de TCR, e a aplicação dos questionários de autoavaliação CSI-Br e LHQ-Br.

A autopercepção da tosse crônica foi utilizada como referência para realização dos pontos de corte. Ela consistiu no autorrelato de tosse por mais de oito semanas, persistente ao tratamento médico e não relacionada a outras condições de saúde (quadro clínico de TCR)^(3)^.

O CSI-Br é um instrumento validado em PB^(12,13)^, composto por 10 itens, que mensuram a gravidade dos sintomas de TC e sua influência em diferentes aspectos da vida do paciente^(16)^. O instrumento possui uma chave de resposta com escala do tipo Likert de cinco níveis, entre zero (nunca) e quatro (sempre). O instrumento possui dois fatores: atividades físicas e sociais, composto pelos itens 2, 3, 4, 5, 8 e 10; e, funcional e psicológico composto pelos itens 1, 6, 7 e 9. Quanto maior o escore do CSI-Br, maior a percepção do paciente sobre a gravidade da tosse e o impacto dos sintomas na qualidade de vida com uma pior condição de saúde autopercebida^(16)^.

O LHQ-Br é um instrumento de autopercepção das sensações laríngeas associadas à hipersensibilidade laríngea^(17)^, validado em PB^(14,15)^. Trata-se de um instrumento composto por 12 itens e uma chave de resposta em escala do tipo Likert entre um (sempre) e sete (nunca). O LHQ-Br possui apenas um fator, calculado pela média dos 12 itens. Quanto maior o escore do LHQ-Br, menor a percepção das sensações laríngeas. Sendo assim, quanto menor a pontuação, pior é considerado o quadro de tosse do paciente^(17)^.

O software estatístico utilizado foi o IBM SPSS Statistics 29.0. Foi determinado o valor do ponto de corte pela análise da área sob a curva com o uso da “Receiver Operating Characteristic” (ROC) curve, a partir da relação entre os maiores valores de sensibilidade e especificidade^(13,14)^. A curva ROC mede a capacidade de discriminação de um teste, ou seja, sua habilidade de distinguir entre indivíduos com e sem uma condição específica. O ponto de corte foi avaliado como classificador utilizando-se o teste Kolmogorov-Smirnov máximo (KS) e o índice de Gini. A AUC varia entre 0 e 1, sendo valores acima de 0,7 considerados bons, entre 0,71 e 0,8 excelentes e abaixo de 0,69 indicativos de discriminação nula^(13,14)^.

## RESULTADOS

O LHQ-Br no escore total apresentou uma AUC de 0,753 (IC 95%: 0,666 a 0,841; p < 0,001), conforme mostram a Figura 1 e a Tabela 1.

**Figura 1:** Curva ROC do Questionário Newcastle de Hipersensibilidade Laríngea (LHQ-Br)

**Tabela 1:** Curva ROC do Questionário Newcastle de Hipersensibilidade Laríngea (LHQ-Br)

Variável	Área	Estatística do teste	p-valor	IC 95%	
				Limite inferior	Limite superior
LHQ-Br	0,753	0,045	0,000	0,666	0,841

Legenda: IC = intervalo de confiança; LHQ-Br = Questionário Newcastle de Hipersensibilidade Laríngea

Obteve-se para o LHQ-Br um índice de Gini de 0,507 e uma métrica de KS de 0,467, com o ponto de corte sugerido em 5,8 e uma qualidade de modelo de 0,67. Esse ponto de corte apresenta sensibilidade de 70,0% e especificidade de 73,4% (1 - especificidade = 0,266), resultando em um índice de Youden de 0,467 (Tabela 2).

**Tabela 2:** Coordenadas da Curva ROC do Questionário Newcastle de Hipersensibilidade Laríngea (LHQ-Br)

Variável	Positivo se maior ou igual a	Sensibilidade	1 - Especificidade	Índice do Youden
LHQ-Br	5,8	0,700	0,266	0,467

Legenda: LHQ-Br = Questionário Newcastle de Hipersensibilidade Laríngea

O CSI-Br no escore total apresentou uma AUC de 0,894 (IC 95%: 0,827 a 0,960; p < 0,001). O fator atividades físicas e sociais obteve uma AUC de 0,832 (IC 95%: 0,752 a 0,912; p < 0,001), e o fator psicológico e funcional teve a maior AUC entre os escores analisados, com valor de 0,898 (IC 95%: 0,837 a 0,959; p < 0,001). Esses resultados podem ser visualizados na Figura 2 e na Tabela 3.

**Figura 2:** Curva ROC do Índice de Severidade da Tosse (CSI-Br)

**Tabela 3:** Área sobre a curva ROC do Índice de Severidade da Tosse (CSI-Br)

Variável	Área	Estatística do teste	p-valor	IC 95%	
				Limite inferior	Limite superior
CSI-Br Total	0,894	0,034	0,000	0,827	0,960
CSI-Br Atividade física e social	0,832	0,041	0,000	0,752	0,912
CSI-Br Psicológico e emocional	0,898	0,031	0,000	0,837	0,959

Legenda: IC = intervalo de confiança; CSI-Br = Índice de Severidade da Tosse

Observou-se que o CSI-Br Total apresentou um índice de Gini de 0,787 e métrica KS de 0,679, com o ponto de corte sugerido em 4,50 e uma qualidade de modelo de 0,83. Esse ponto de corte apresenta sensibilidade de 84,9% e especificidade de 83,0%, com índice de Youden de 0,679. O fator CSI-Br Physical and Social Activities mostrou índice de Gini de 0,663 e métrica KS de 0,638, com ponto de corte em 1,50 e uma qualidade de modelo de 0,84. Esse ponto de corte apresenta sensibilidade de 80,8% e especificidade de 83,0%, resultando em um índice de Youden de 0,638. O fator CSI-Br Psychological and Functional teve um índice de Gini de 0,796 e métrica KS de 0,661, com o ponto de corte de 4,50 e qualidade de modelo de 0,75. Com esse ponto de corte obtém-se uma sensibilidade de 76,7% e especificidade de 89,4%, com índice de Youden de 0,661 (Tabela 4).

**Tabela 4:** Coordenadas da Curva ROC do Índice de Severidade da Tosse (CSI-Br)

Variável	Positivo se maior ou igual a	Sensibilidade	1 - Especificidade	Índice do Youden
CSI-Br Total	4,50	0,849	0,170	0,679
CSI-Br Atividade física e social	1,50	0,808	0,170	0,638
CSI-Br Psicológico e emocional	4,50	0,767	0,106	0,661

Legenda: CSI-Br = Índice de Severidade da Tosse

## DISCUSSÃO

A avaliação clínica fonoaudiológica da TCR utiliza testes clínicos e procedimentos de autoavaliação, além de utilizar avaliações vocais como complementares^(10)^. Dentre esses procedimentos, a autoavaliação é importante, visto que mensura dados que não podem ser obtidos por nenhuma outra forma e avaliação clínica, além de mostrar a influência do quadro clínico no dia a dia do paciente^(12,14)^. Por isso, ela tem ganhado mais espaço na avaliação fonoaudiológica, sendo clinicamente considerada como uma avaliação importante para planejar o manejo e analisar a possibilidade de alta fonoaudiológica do paciente de TCR.

A validação dos questionários CSI-Br^(13)^ e LHQ-Br^(15)^, aliados à determinação de seus respectivos pontos de corte, possibilita conhecer a capacidade dessas ferramentas para discriminar os indivíduos com e sem TCR. Essa característica é especialmente relevante no contexto da prática baseada em evidências, pois permite aos profissionais de saúde utilizarem instrumentos válidos e específicos para avaliar a gravidade dos sintomas e o impacto funcional da TCR na vida dos pacientes^18.19^.

Para compreender os pontos de corte, é importante retomar os construtos dos instrumentos: o CSI-Br^(12,13)^ avalia a percepção sobre o impacto da tosse crônica na vida do indivíduo^(16)^, e o LHQ-Br^(14,15)^ avalia a percepção sobre os sintomas relacionados à hipersensibilidade laríngea^(17)^.

A AUC varia entre 0 e 1, sendo que valores 1,0 e 0,9 são considerados excelentes^(20)^, entre 0,9 e 0,8 são considerados bons^(20)^, entre 0,8 e 0,7 são considerados razoáveis^(20)^, entre 0,7 e 0,6 são considerados fracos^(20)^, e abaixo de 0,5 é considerado falha, ou seja, não discrimina melhor que o acaso^(20)^. O LHQ-Br no escore total apresentou uma AUC com valores razoáveis^(20)^, enquanto o CSI-Br foi considerado bom, em todos os fatores e no escore total, sendo que o fator físico e funcional teve a maior AUC entre as medidas.

De acordo com esses índices, ambos os instrumentos tiveram valores aceitáveis, mas o CSI-Br apresentou desempenho mais robusto em todas as métricas analisadas, demonstrando melhor capacidade discriminativa. Os fatores específicos do instrumento, "atividades físicas e sociais" e "psicológico e funcional", fornecem uma visão multidimensional do impacto da TCR, o que pode auxiliar no planejamento de intervenções personalizadas e individualizadas. Este resultado corrobora a literatura que destaca a importância de instrumentos de autoavaliação com abordagens específicas para diferentes aspectos da vida dos pacientes com TCR^(16,17)^.

O LHQ-Br^(15)^ apresentou sensibilidade e especificidade moderadas e equilibradas. Além disso, os índices de Youden, Gini e KS confirmaram um modelo com discriminação moderada. Isso indica que seu ponto de corte pode ser útil para triagem, permitindo identificar pacientes com possíveis sintomas de hipersensibilidade laríngea, e para apoio diagnóstico, auxiliando a reduzir os falsos positivos^(21)^. Contudo, seu desempenho deve ser interpretado como aceitável e complementar, não devendo ser utilizado de forma isolada para confirmar a presença de sintomas de hipersensibilidade laríngea nos pacientes.

Por sua vez, o CSI-Br^(13)^ apresentou-se sensível e específico. Os índices de Youden, Gini e KS confirmaram boa a excelente capacidade discriminativa, tanto no escore total quanto nas subescalas, caracterizando um desempenho consistente, classificando o modelo como bom a excelente em todas as métricas avaliadas. Ele oferece um ponto de corte eficaz tanto para identificar os pacientes com sintomas compatíveis com TCR, quanto para minimizar falsos positivos. Isso o torna uma ferramenta útil para triagem inicial de pacientes com suspeita de TCR e para apoio ao processo diagnóstico^(21)^. Essa característica é particularmente relevante no contexto clínico, uma vez que a identificação precoce de pacientes com sintomas compatíveis com TCR pode favorecer um manejo terapêutico mais assertivo.

Os valores de sensibilidade e especificidade obtidos foram relativamente elevados e próximos entre si, situando-se em nível moderado para o LHQ-Br e em nível elevado para o CSI-Br. Esse achado é relevante, porque ambos apresentam desempenho adequado dentro das dimensões que se propõem a avaliar, porém, a proximidade dos valores não deve ser interpretada como equivalência entre os instrumentos. Isso porque os instrumentos analisam construtos distintos: o LHQ-Br, como marcador da autopercepção das sensações laríngeas associadas à hipersensibilidade laríngea^(12,13,16)^, e o CSI-Br, como medida da gravidade dos sintomas de tosse crônica e de seu impacto em diferentes aspectos da vida do paciente^(14,15,17)^.

O LHQ-Br, embora apresente valores satisfatórios, mostrou uma ligeira redução na sensibilidade e especificidade em comparação à sua versão original validada em inglês^(16)^. Essa diferença pode ser atribuída às especificidades culturais do PB e às diferenças no estabelecimento do ponto de corte. O CSI-Br^(13)^ manteve boa acurácia em relação ao protocolo original validado em inglês^(16)^, que também reportou altos valores de AUC, geralmente acima de 0,85, e índices elevados de sensibilidade e especificidade.

Ao analisar os dados de forma conjunta, sem comparação direta entre os instrumentos, observa-se que uma possível limitação do LHQ-Br^(15)^, em relação ao CSI-Br^(13)^, está em sua chave de resposta mais extensa — uma escala do tipo Likert com sete pontos. Essa característica o diferencia da maioria dos instrumentos de autoavaliação utilizados em contextos clínicos, que geralmente adotam escalas mais concisas, o que pode impactar a compreensão e a consistência das respostas por parte dos pacientes.

Adicionalmente, é importante considerar que o presente estudo contou com uma amostra composta exclusivamente por pacientes com TCR, enquanto o escopo de aplicação da ferramenta para hipersensibilidade laríngea é mais abrangente. Essa diferença pode influenciar a generalização dos achados e destaca a necessidade de futuras investigações com populações mais heterogêneas dentro do espectro da hipersensibilidade laríngea.

O ponto de corte do LHQ-Br na linguagem original^(17)^ é 17,1 e no presente estudo foi de 5,8. Essas divergências podem ser atribuídas à estruturação diferente dos instrumentos. No processo de validação em PB^(15)^ foram eliminados dois itens no LHQ-Br, e a estrutura foi unifatorial. O escore, por sua vez, foi calculado pela média dos itens^(15)^. O instrumento internacional é composto por 14 itens distribuídos em três fatores (sensibilidade laríngea, sensação de desconforto, sensação de pressão)^(17)^. Seu escore por fator é de média, e o total é a somatória da média dos três fatores^(17)^. Porém, considerando ser unifatorial a versão em PB^(15)^, ao dividir por três o ponto de corte internacional de 17,1, obtém-se 5,7, valor muito semelhante ao ponto de corte brasileiro de 5,1.

O CSI-Br possui um ponto de corte de 3 na língua original^(16)^ e de 4,5 em PB. A versão brasileira^(13)^ possui dez itens, divididos em dois fatores (atividades físicas e sociais, psicológico e funcional), o que reflete uma preocupação com as dimensões específicas da qualidade de vida, adaptadas à percepção cultural dos sintomas, enquanto a versão internacional possui dez itens tratados como um único escore total^(16)^. Além disso, vale destacar que o ponto de corte internacional foi estabelecido com base na média da diferença entre indivíduos com e sem TCR, acrescendo-se a esse valor a pontuação de dois desvios padrões da média^(16)^. Acredita-se que a diferença nos procedimentos de estabelecimento do ponto de corte justifique a divergência para o ponto de corte em PB.

Este estudo destaca a relevância da autoavaliação na prática clínica, principalmente em condições que possuem componentes subjetivos significativos, como a TCR. A capacidade dos pacientes de relatar suas percepções e experiências em relação aos sintomas contribui para a definição do diagnóstico e permite uma abordagem mais centrada no paciente. Dessa forma, a definição dos pontos de corte representa um avanço importante ao transformar dados subjetivos em informações clinicamente interpretáveis, contribuindo para melhorar a tomada de decisão clínica. Sendo assim, os pontos de corte dos instrumentos autoavaliativos fonoaudiológicos também contribuem para o progresso científico dentro dos estudos relacionados à TCR.

Ainda que o presente estudo tenha fornecido evidências robustas sobre a aplicabilidade e utilidade clínica do CSI-Br e do LHQ-Br, é importante considerar que diante das condições específicas do contexto brasileiro, no qual há limitações de acesso a tecnologias diagnósticas objetivas para a TCR. Essa realidade torna os instrumentos estudados ainda mais relevantes, mas também representa uma limitação, uma vez que a referência utilizada foi a autopercepção do paciente e não uma avaliação objetiva do diagnóstico da TCR. Além disso, o estudo apresenta como limitação a distribuição desigual entre os grupos, o que pode reduzir a precisão da estimativa da especificidade. Essa limitação foi mitigada pela apresentação dos intervalos de confiança de 95%, que expressam a real incerteza das estimativas e permitem interpretação adequada dos achados.

## CONCLUSÃO

Os pontos de corte identificados foram de 5,8 para o LHQ-Br e 4,5 para o CSI-Br, com valores específicos de 1,5 para o domínio físico e social, e 4,5 para o domínio psicológico e funcional. Os resultados indicam que o LHQ-Br apresenta aplicabilidade moderada para triagem inicial e apoio diagnóstico de pacientes com sensações laríngeas associadas à hipersensibilidade laríngea. O CSI-Br demonstrou desempenho mais robusto, com utilidade tanto para triagem inicial quanto como instrumento de apoio no processo diagnóstico, ao mensurar a gravidade dos sintomas de tosse crônica refratária e sua influência em diferentes aspectos da vida do paciente. Esses achados reforçam a aplicabilidade de ambos os instrumentos, que contribuem de maneira distinta conforme seus construtos, mas são ambos relevantes para o reconhecimento precoce e manejo adequado de pacientes com TCR.
